# Clinical characteristics and 12-month outcomes of patients with valvular and non-valvular atrial fibrillation in Kenya

**DOI:** 10.1371/journal.pone.0185204

**Published:** 2017-09-21

**Authors:** Tecla M. Temu, Kathleen A. Lane, Changyu Shen, Loise Ng'ang'a, Constantine O. Akwanalo, Peng-Sheng Chen, Wilfred Emonyi, Susan R. Heckbert, Myra M. Koech, Imran Manji, Matteo Vatta, Eric J. Velazquez, Jennifer Wessel, Sylvester Kimaiyo, Thomas S. Inui, Gerald S. Bloomfield

**Affiliations:** 1 Department of Medical Microbiology, University of Nairobi College of Health Sciences, Nairobi, Kenya; 2 Department of Biostatistics, Indiana University School of Medicine, Indiana University, Indianapolis, IN, United States of America; 3 The Smith Center for Outcomes Research in Cardiology, Beth Israel Deaconess Medical Center and Harvard Medical School, Boston, MA, United States of America; 4 Department of Medicine, Duke Clinical Research Institute and Duke Global Health Institute, Duke University, Durham, NC, United States of America; 5 Division of Medicine, Moi Teaching and Referral Hospital, Eldoret, Kenya; 6 Academic Model Providing Access to Healthcare (AMPATH), Eldoret, Kenya; 7 Department of Medicine and Molecular Genetics, Indiana University, Indianapolis, IN, United States of America; 8 Department of Medicine, Indiana University, Indianapolis, IN, United States of America; 9 Department of Epidemiology, University of Washington, Seattle, WA, United States of America; 10 Department of Medicine, School of Medicine, College of Health Sciences, Moi University, Eldoret, Kenya; Nagoya University, JAPAN

## Abstract

**Background:**

Atrial fibrillation (AF) is a major contributor to the global cardiovascular disease burden. The clinical profile and outcomes of AF patients with valvular heart diseases in sub-Saharan Africa (SSA) have not been adequately described. We assessed clinical features and 12-month outcomes of patients with valvular AF (vAF) in comparison to AF patients without valvular heart disease (nvAF) in western Kenya.

**Methods:**

We performed a cohort study with retrospective data gathering to characterize risk factors and prospective data collection to characterize their hospitalization, stroke and mortality rates.

**Results:**

The AF patients included 77 with vAF and 69 with nvAF. The mean (SD) age of vAF and nvAF patients were 37.9(14.5) and 69.4(12.3) years, respectively. There were significant differences (p<0.001) between vAF and nvAF patients with respect to female sex (78% vs. 55%), rates of hypertension (29% vs. 73%) and heart failure (10% vs. 49%). vAF patients were more likely to be taking anticoagulation therapy compared to those with nvAF (97% vs. 76%; p<0.01). After 12-months of follow-up, the overall mortality, hospitalization and stroke rates for vAF patients were high, at 10%, 34% and 5% respectively, and were similar to the rates in the nvAF patients (15%, 36%, and 5%, respectively).

**Conclusion:**

Despite younger age and few comorbid conditions, patients with vAF in this developing country setting are at high risk for nonfatal and fatal outcomes, and are in need of interventions to improve short and long-term outcomes.

## Introduction

Atrial fibrillation (AF) is the most common cardiac arrhythmia affecting over 33 million people worldwide [1,2]. AF is associated with increased risk of stroke, heart failure and mortality [3–5]. Variations in AF incidence and complications have been observed by age, gender and race [5]. Although previous studies in high-income countries have reported a lower prevalence of AF in people of African descent compared to those of European ancestry [6], AF is becoming increasingly common in sub-Saharan Africa [7]. Between 1990 and 2010, AF represented the largest relative increase in cardiovascular disease burden in sub-Saharan Africa (SSA) going from 0.7% to 1% [7], while rates of other cardiovascular diseases such as ischemic heart diseases increased at a much lower rate over the same period. Nonetheless, there remains a paucity of data on clinical characteristics and outcomes associated with AF in this region.

Although rheumatic heart disease (RHD) is relatively rare in high-income countries, it remains a major risk factor of AF in low-income countries where it is present in nearly one out of five patients with AF [8]. Patients with RHD tend to be younger and more often women of reproductive age [9]. It is estimated that 8% of strokes in low and middle-income countries could be due to complications of RHD [10]. Despite the magnitude of the problem, these patients are usually excluded from research studies related to AF, particularly larger clinical trials of anticoagulation therapy. As a result, there is sparse data assessing characteristic and outcomes of patients with valvular diseases and AF. And, it is still unknown if AF patients with or without valvular diseases have a similar risk of stroke, mortality or hospitalization. Given the rapid increase in the prevalence of AF in SSA, it is imperative to understand the clinical characteristics and outcomes of AF patients, in particular the differences between valvular and non-valvular AF populations. In this study, we evaluated clinical characteristics, treatment patterns and 12-month outcomes of valvular AF patients in comparison to the non-valvular AF patients enrolled in the Study of Genetics of Atrial Fibrillation in an African Population (SIGNAL) study.

## Methods

### Study population and design

As previously described in detail [11], SIGNAL is a study designed to determine characteristics, treatment patterns, 12-month outcomes and genetic associations in patients with AF in western Kenya. The study consisted of retrospective data-gathering during study enrollment to characterize the differences between valvular and non-valvular AF patients, and prospective data collection 12-month after enrollment to characterize their hospitalization, stroke and mortality rates. We enrolled adult patients (>18 years) with a diagnosis of AF who presented to the cardiology clinic, inpatient wards, medical outpatient clinic, anticoagulation clinic or the cardiac noninvasive diagnostic unit at Moi Teaching and Referral Hospital (MTRH) in Eldoret, Kenya. Patients were enrolled consecutively. This site was chosen because of its broad mix of patients including a mix of urban middle class, urban poor and rural populations and presence of an NHLBI-supported Cardiovascular Center of Excellence in research at MTRH [12]. The study sample included two groups of AF patients, those with valvular AF (vAF) and those with non-valvular AF (nvAF). Both prevalent and incident cases of AF were included, and no distinction was made according to duration of AF episodes. Atrial Fibrillation had to be recorded on at least one 12-lead electrocardiogram (ECG). Patients with known genetic syndromes, congenital heart defects, and other severe illnesses precluding ECG or echocardiogram were excluded. Detailed inclusion and exclusion criteria have been published previously [11].

### Study procedure

The Institutional Research and Ethics Committee of Moi University School of Medicine, and the Institutional Review Boards of Indiana University and Duke University approved the study. All patients provided written informed consent. Medical history, physical examination, biochemical analysis, 12-lead electrocardiogram and an echocardiogram were performed at baseline. The diagnosis of nvAF was based on ruling out significant valvular heart disease through a combination of history, echocardiogram, ECG, and clinical findings. Data were collected using a standardized case report form. We calculated each participant’s CHADS_2_ score to assess stroke risk in which one point is assigned for each of the following characteristics: a history of congestive heart failure, hypertension, age ≥75 years, or diabetes mellitus, and 2 points are assigned for a history of stroke or transient ischemic attack [13]. Sufficient data were not available to calculate a CHA_2_DS_2_-VASc score. The 12-month follow-up was carried out by telephone or in-person interview.

### Outcomes

The main study outcomes were all-cause mortality, stroke and cardiac hospitalization. Combined adverse events (CAE) was defined as a composite of all-cause mortality, stroke and cardiac hospitalization. Death and hospitalization were determined as reported by the participant or the relatives of the participant during follow-up by telephone and, when possible, medical records were also reviewed. Deaths were further classified as being cardiovascular (e.g. sudden cardiac death, heart failure, stroke and other vascular causes) or non-cardiovascular due to other specified causes (e.g. malignancy, infection etc.). Stroke was also determined as reported by the relatives or patients and, when possible, medical records were also reviewed. Mortality was treated as a time-to-event outcome while other outcomes were treated as binary.

### Statistical analysis

Baseline clinical characteristics including demographics, medical history, and medical therapies were compared between vAF and nvAF patients. Descriptive statistics for continuous variables were expressed as means (standard deviation [SD]) or median (interquartile range [IQR]) while those for categorical variables were expressed as frequencies and percentages unless otherwise specified. Comparisons of baseline characteristics and outcomes between vAF and nvAF patients were conducted using Fisher’s exact test for categorical variables and with t-tests or Wilcoxon rank-sum tests for continuous variables. Survival curves (all-cause mortality) were created for the two groups of AF patients analyzed using the Kaplan–Meier method, and compared using the log-rank test. A p < 0.05 was considered to be statistically significant. Statistical analysis was performed using SAS version 9.4. See supporting information for the minimum dataset underlying our results.

## Results

### Baseline characteristics of SIGNAL patients

**Fig 1:**
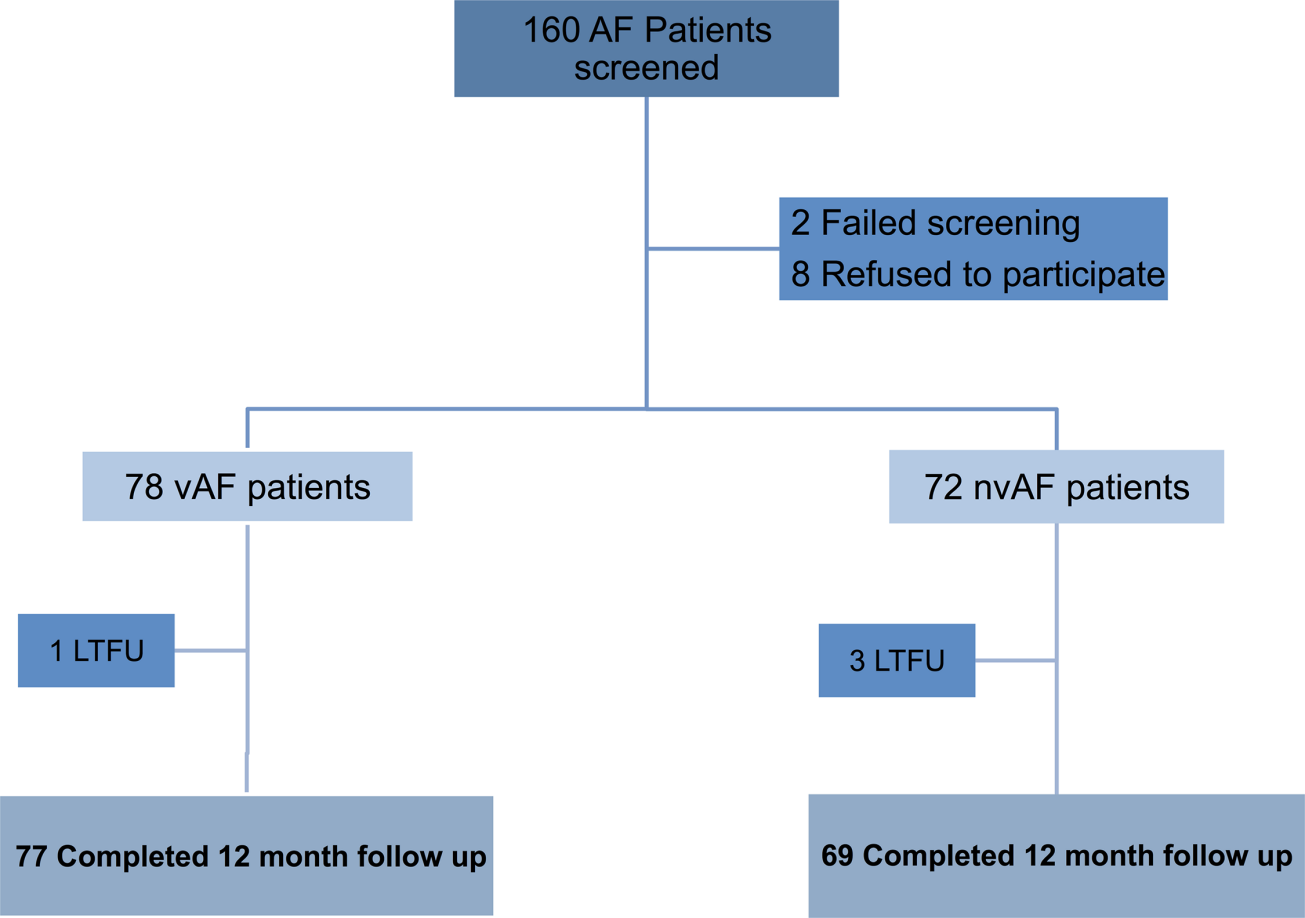
Flow diagram of enrollment and12-months follow-up. Legend. AF = atrial fibrillation, LTFU = lost to follow up.

From October 2013 through July 2014, a total of 160 consecutive AF patients were screened. Of these, 150 AF patients were enrolled in the study. The median duration of the follow-up period was 12 (12–14) months. Follow up data was available for 77 vAF and 69 nvAF patients with 4 patients lost to follow-up, yielding 146 AF patients (97%) included in the analysis (Fig 1). The baseline characteristics of the two groups are shown in Table 1. All patients with vAF reported a history of rheumatic fever. Non-mutually exclusive, valvular heart diseases subtypes included mitral regurgitation (86%), moderate to severe mitral stenosis (53%) and aortic regurgitation (49%). vAF compared to nvAF patients were significantly younger and more often females. They were less likely to have hypertension, heart failure, previous myocardial infraction, history of alcohol consumption and tobacco use and had significantly lower systolic blood pressure. Those with vAF had significantly larger left atrial volume than the nvAF patients. Other lab data including creatinine were comparable between the two groups.

**Table 1:** Baseline characteristics of atrial fibrillation patients.

			vAF (n = 77)	nvAF (n = 69)	p value (vAF vs. nvAF)
Demographics					
	Female		60 (77.9)	38 (55.1)	0.005
	Age, yrs		37.9 ± 14.5	69.4 ± 12.3	<0.001
	Age groups				<0.001
		18–40	48 (62.3)	2 (2.9)	
		41–59	22 (28.6)	15 (21.7)	
		60–74	7 (9.1)	26 (37.7)	
		≥75	0 (0.0)	26 (37.7)	
Medical history					
	Heart failure		6 (9.5)	33 (48.5)	<0.001
	Hypertension		22 (28.6)	50 (72.5)	<0.001
	Stroke		18 (23.4)	18 (26.1)	0.85
	DM		1 (1.3)	6 (8.7)	0.052
	RHD		77 (100)	0 (0.0)	<0.001
	Thyroid Disease		2 (3.0)	3 (5.0)	0.67
	MI		4 (5.4)	8 (11.8)	0.23
	Dsylipidemia		7 (9.2)	14 (20.3)	0.06
	COPD		2 (2.7)	10 (14.9)	0.01
	Heart surgery		16 (20.8)	0 (0.0)	<0.001
	HIV		2 (2.6)	4 (5.9)	0.42
	Sleep apnea		4 (5.3)	5 (7.4)	0.74
	Tobacco use		6 (7.8)	26 (37.7)	<0.001
	Alcohol drinking		12 (15.6)	40 (58.0)	<0.001
Other characteristics					
	AF duration, yrs		6.6 ± 7.5	3.4 ± 6.6	0.007
	SBP, mm Hg		114.9 ± 17.8	126.4 ± 23.0	0.001
	DBP, mm Hg		73.1 ± 12.8	75.9 ± 16.2	0.25
	Heart rate, beats/min		84.5 ± 21.1	80.6 ± 22.8	0.29
	CHADS_2_ score		Na	2.2 ± 1.3	
	BMI, kg/m^2^		22.0 ± 11.0	23.7 ± 5.1	0.23
NYHA Class					0.19
	I		56 (73.7)	45 (65.2)	
	II		10 (13.2)	11 (15.9)	
	III		7 (9.2)	4 (5.8)	
	IV		3 (3.9)	9 (13.0)	
Lab values					
BNP, pg/mL (IQR)			745.2 (37.3–2543)	1288.5 (29.2–3895.0)	0.20
HbA1c, %			5.8 ± 0.6	6.0 ± 0.7	0.01
Creatinine, mmol/L			68.0 ± 18.7	76.0 ± 33.9	0.08
INR			2.4 ± 0.9	2.4 ± 0.9	0.81
hs-CRP, mmol/L			5.4 ± 7.4	8.8 ± 15.7	0.10
Echocardiographic characteristics					
LV mass, g			158.3 ± 68.5	148.2 ± 57.3	0.38
LVEF, %			45.5 ± 10.7	43.5 ± 12.9	0.47
LA volume, cm^3^			247.2 ± 142.4	111.4 ± 49.4	<0.001

Values are in n (%), median (interquartile range) or mean ± SD.

AF, Atrial fibrillation; nvAF, Non-valvular AF; vAF, Valvular AF; RHD, Rheumatic heart disease; COPD, chronic obstructive pulmonary disease; SBP, Systolic BP; DBP, Diastolic BP; DM, Diabetes mellitus; MI, Myocardial infraction; hs-CRP, High-sensitivity C-reactive Protein; BNP, B-type Natriuretic Peptide; LVEF, Left ventricular ejection fraction; LA, left atrial; LV, Left ventricular; na, not applicable.

### Treatment characteristics

The most frequent medications used in among AF patients were diuretics (76.7%), beta-blockers (49.3%) and digoxin (35.6%). Prescription of ACE inhibitors, beta-blockers and diuretics were similar between the two groups (Table 2). Information on amiodarone and calcium blockers drug treatment and history of electrical cardioversion, while available in our setting, were not recorded. Treatments to achieve and maintain sinus rhythm such as catheter ablation therapy are not available in our setting. None of the participants received direct oral anticoagulant therapy, as these medications are not generally available to patients in this setting. 16 vAF patients had undergone surgery for valve replacement or repair, valve replacement (12) or repair (4). Nine patients had mitral valve replacement alone, one had aortic valve replacement and one had combined aortic and mitral valve replacement. All replaced valves were mechanical, and all repairs were mitral valve repairs. Significantly more vAF patients than nvAF received anticoagulation therapy (97.4% vs. 91.3%, p< 0.01). However, most of nvAF patients with CHADS_2_ scores ≥2 received warfarin (79.1%). All vAF participants who underwent valve replacement or therapy were on warfarin. INR measurements were obtained for all AF patients on warfarin therapy at enrollment and the overall mean was 2.4 (0.9). Of the vAF and nvAF patients, 29.3% vs. 28.8% were in the sub-therapeutic range (<2.0), 52.0% vs. 55.9% in the therapeutic range (2.0–3.0) and 18.7% vs. 15.2% above the therapeutic range (>3.0), respectively. None of the participants received direct oral anticoagulant therapy, as these medications are not generally available to patients in this setting.

**Table 2:** Medication use before study entry for atrial fibrillation patients.

	vAF (n = 77)	nvAF (n = 69)	p value (vAF vs. nvAF)
Warfarin	75 (97.4)	56 (81.2)	0.002
Aspirin	0 (0.0)	7 (10.1)	0.004
Digoxin	32 (41.6)	20 (29.0)	0.12
ACE inhibitor	21 (27.3)	24 (34.8)	0.37
ARB	3 (3.9)	18 (26.1)	<0.001
Diuretic	56 (72.7)	56 (81.2)	0.25
Beta-blocker	42 (54.5)	30 (43.5)	0.19

Values are n (%), ACEI, angiotensin-converting enzyme inhibitors; ARB, angiotensin receptors blockers.

Other abbreviations as in Table 1

### Mortality and cardiovascular outcomes

**Fig 2:**
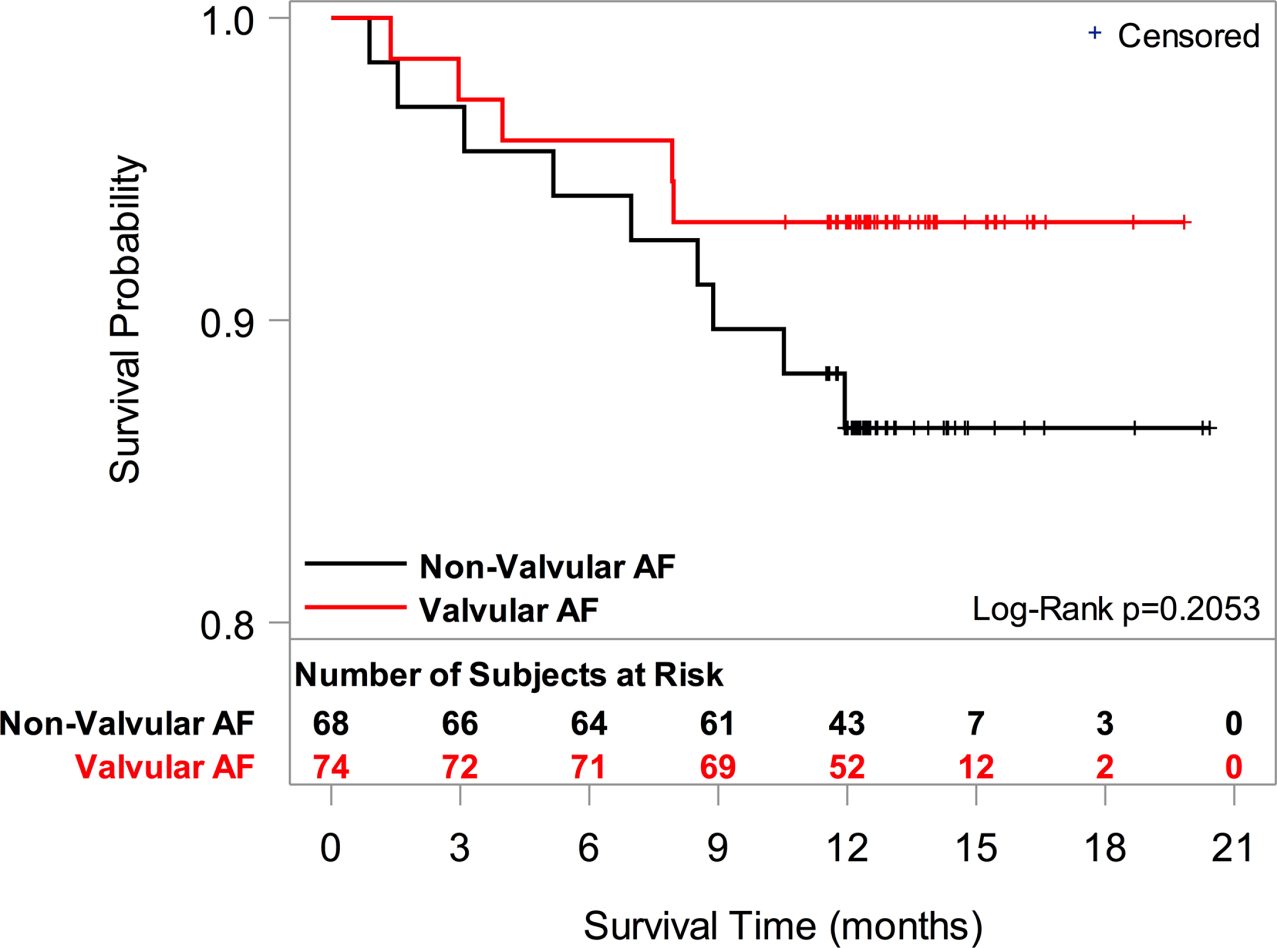
Kaplan-meier survival curve for atrial fibrillation patients. Legend. AF = atrial fibrillation.

Table 3 shows 12-month event rates for each group where death is treated as a binary outcome. Overall incidence of mortality, stroke, and cardiac related hospital admissions were high in both groups without statistically significant differences. There were a total of 18 deaths (14 cardiovascular, 1 non-cardiovascular, 3 with unknown cause of death), 7 strokes and 47 hospitalizations (39 for cardiac reasons) during the follow-up period. There were (8 vs. 10; p = 0.46) deaths, (4 vs. 3; p = 1.00) stroke and (19 vs.20; p = 0.71) cardiac related hospitalization for vAF and nvAF patients, respectively. There were no reported deaths among vAF who underwent valve repair or replacement. Fig 2 presents the Kaplan-Meier survival curve (i.e., time-to-event analysis) for the two AF patient groups.

**Table 3:** Comparison of outcomes of atrial fibrillation patients.

Outcomes		vAF (n = 77)	nVAF (n = 69)	p-value (vAF vs. nvAF)
All-cause mortality		8 (10.4)	10 (14.5)	0.46
Cause of death				0.40
	Cardiovascular death	5 (62.5)	9 (90.0)	
	Non-Cardiovascular death	1 (12.5)	0 (0.0)	
	Unknown cause	2 (25.0)	1 (10.0)	
Stroke ^†^		4 (5.4)	3 (4.5)	1.00
Hospitalization		23 (31.1)	24 (35.8)	0.59
Cardiac-hospitalization ^‡^		19 (25.7)	20 (29.9)	0.71
Combined adverse events		24 (31.2)	21 (30.4)	1.00

Values are n (%). Combined adverse events (composite of all-cause of mortality, stroke and cardiac hospitalization).

Other abbreviations as in Table 1.

^†^N = 140

^‡^N = 141

## Discussion

We demonstrated that vAF patients, who make up a large proportion of AF patients in SSA, are a cohort of relatively young patients, predominantly women, less likely to have the traditional clinical characteristics related to poor outcomes including heart failure, hypertension, diabetes and previous myocardial infraction, compared to their nvAF counterparts. Despite differences in clinical profile and significantly younger age, the risk of death, stroke occurrence and hospitalizations were similar to those of nvAF patients. This subgroup of AF patients with valvular heart diseases attributed to rheumatic heart diseases (RHD) represents a group with high morbidity and mortality, in need of interventions to improve outcomes.

The average age of vAF patients in this study was 38 years, significantly younger compared to our nvAF cohort as well as the AF patients with RHD who participated in the RE-LY cohort study [52 years) [8]. In addition to being younger, they were predominantly women, with a large majority of childbearing age. Prior literature concurs that most RHD patients are women [9]. Given the potential complications associated with maternal cardiovascular diseases and the unique challenges for stroke prevention in pregnancy, the ability to both counsel patients prior to pregnancy and appropriately manage these patients is of vital importance. Unfortunately, the majority of the women with RHD in low-income countries are not aware of the pre-existing heart disease until pregnancy. In this study, one fifth of women with vAF reported a previous stroke. Our results highlight the need for community based screening and prevention of RHD in low-middle income countries.

The prevalence of cardiovascular disease risk factors, comorbid conditions, tobacco and alcohol use were all significantly higher in nvAF patients than in vAF patients. Consistent with previous reports, hypertension was the most common AF risk factor among the nvAF patients (72%) but was less common in patients with vAF (28%) [5,14–17]. Diabetes prevalence was strikingly low in both groups consistent with previous findings in patients with AF from SSA [8]. With the expected aging of the population in SSA, we expect to see a surge in cardiovascular risk factors, particularly hypertension, which portends more risk for fatal outcomes. Thus, in addition to rate control and anticoagulation therapy, there is a great need for comprehensive strategies for prevention, treatment and control of hypertension in both groups of AF patients.

History of stroke in our AF cohorts was common and greater than seen in previous reports from other countries in SSA [14–17]. Anticoagulation is recommended for all patients with AF and RHD [18]. Oral anticoagulation therapy use was higher than reported in several worldwide registries (4%-79%) [19–21]. However stroke occurred in 5.4% of the vAF cases during 12 months’ follow-up, despite high use of warfarin, younger age and few cardiovascular risk factors. The stroke rate for vAF patients is similar to that reported in the RE-LY global registry of AF for AF patients with RHD (4.2%), where they also found no significant difference in stroke risk between AF patients with and without RHD [4]. Possible factors that may contribute to increased risk of stroke in this young group of vAF patients are co-morbid conditions including heart failure, genetic predisposition, left atrial enlargement and potentially low time in therapeutic range for warfarin. A high proportion of AF patients were on anticoagulation therapy. All AF patients with indication for anticoagulation seeking care at Moi Teaching and Referral Hospital, a second largest referral hospital in Kenya are routinely referred to anticoagulation clinic, an important feature of AMPATH care introduced by our clinical pharmacists and very remarkable in the SSA context. However, less than two thirds of these patients had INR in the therapeutic range. Our results highlight the importance of affordable high-quality anticoagulation therapy and research into the safety and efficacy of novel oral anticoagulants in this high-risk group. Data on stroke incidence in vAF cases is not widely reported from SSA. Forthcoming data from the Global Rheumatic Heart Disease Registry should provide further context for our findings [22].

Population-based data and modeling studies demonstrate that AF confers an excess risk of cardiovascular morbidity and mortality [2,4,23]. However, owing to paucity of data, uncertainty remains on the consequences of vAF in the SSA region. In our analysis, almost one-third of patients with vAF reported cardiac related hospitalization, while 10% died during 12 months’ follow-up. No significant difference in mortality and hospitalization were detected between the vAF and nvAF cohort. High rates of adverse events in the vAF patients are likely due to complications of AF and the fact that these patients tend to present for care at advanced stages of valvular disease—thereby increasing the probability of severe complications such as heart failure and stroke. Due to a small sample size and short duration of follow-up, we did not have sufficient events to assess the determinants of mortality and cardiac related hospitalization. Future studies in larger cohorts from this region should be considered to confirm these findings.

### Study limitations

There are several limitations to this study worth highlighting. This study enrolled a convenience sample of patients seeking care at tertiary clinics in western Kenya and it is uncertain whether the study results can be generalized to other populations. Data on the classification of AF (paroxysmal, persistent, or chronic) was not available hence, we cannot comment on differences in the prognosis of these AF subsets. Onset of AF was by patient self-report which was not able to be objectively confirmed. Due to a small sample size and short duration of follow-up, we did not have sufficient number of events to adjust for covariates and we could not calculate adjusted rates for stroke, mortality or cardiac hospitalization.

## Conclusion

Despite their relative youth, patients in sub-Saharan Africa with vAF are at increased risk for adverse cardiovascular events. RHD may be as important as other well-established factors in predicting adverse cardiovascular outcomes of AF patients in this region. Strategies aimed at preventing cardiovascular disease risk factors and RHD are needed.

## Supporting information

S1 File Dataset underlying the study results. (XLSX)
